# Predictors of Quality of Life in Patients With Myocardial Infarction Combined With Dyslipidemia

**DOI:** 10.3389/fpubh.2021.713480

**Published:** 2021-10-06

**Authors:** Mengran Zhang, Pingyu Chen, Yan Zhang, Xi Su, Jiyan Chen, Biao Xu, Jianhong Tao, Zhen Wang, Hongchao Li, Aixia Ma

**Affiliations:** ^1^School of International Pharmaceutical Business, China Pharmaceutical University, Nanjing, China; ^2^Department of Cardiology, Peking University First Hospital, Beijing, China; ^3^Department of Cardiology, Wuhan Asia Heart Hospital, Wuhan, China; ^4^Department of Cardiology, Guangdong Provincial People's Hospital, Guangzhou, China; ^5^Department of Cardiology, Nanjing Drum Tower Hospital, Nanjing, China; ^6^Department of Cardiology, Sichuan Provincial People's Hospital, Chengdu, China; ^7^Department of Cardiology, Zhongshan Hospital, Fudan University, Shanghai, China

**Keywords:** myocardial infarction, dyslipidemia, health-related quality of life, predictors, real-world data, China

## Abstract

**Background:** Dyslipidemia is an important risk factor for myocardial infarction (MI). This study aimed to examine the health-related quality of life (HRQoL) and its predictors of patients with MI combined with dyslipidemia in China.

**Methods:** Information on patients' sociological characteristics, lifestyle, clinical characteristics, and quality of life were collected by electronic medical records and questionnaires. Tobit regression model was used to investigate the predictors of quality of life.

**Results:** There were 756 patients responded. The average EQ-5D score of all the patients was 0.95 (SD: 0.11). For all patients, factors such as age, high-fat and high-cholesterol diet, sports, family history of dyslipidemia, history of peripheral artery disease significantly affected HRQoL.

**Conclusions:** Post-discharge care of the elderly group should be paid more attention to and suggestions on the healthy lifestyle (fat control) of the patients should be encouraged to improve the quality of life of these population.

## Introduction

Coronary heart disease (CVD) is caused by coronary artery luminal stenosis or occlusion. A survey of the World Health Organization showed that 17.9 million people die each year from CVD (1). Myocardial infarction (MI) is the most serious type of coronary heart disease. The World Bank estimates that the number of people with MI in China will rise to 23 million in 2030 (2). The disease burden of MI is relatively heavy. The mortality rate of acute myocardial infarction (AMI) raised sharply from 14.04 per 100,000 people in 2004 to 64.25 in 2014 (3). Dyslipidemia is a strong predictor of MI (4, 5), Patients should strictly control their low-density lipoprotein-cholesterol levels (LDL-C) (6). In China, the prevalence of Dyslipidemia in Chinese people aged ≥18 years was 40.40% (7). Such a high prevalence of dyslipidemia may increase the risk of MI, and it is necessary to control blood lipids, especially in patients with MI.

Health-related quality of life (HRQoL) is generally considered as a multidimensional assessment of the patients' overall physiological and psychological function, which can reflect the influence of different stages of disease and treatment measures on the patients' health status (8). HRQoL can also be combined with time to calculate quality-adjusted life years (QALYs) to compare the economics of different interventions in pharmacoeconomic evaluation. In recent years, although the prognosis and survival rate of MI have been significantly improved, the motor ability of patients after acute MI is impaired (9), and quality of life is also negatively affected (mobility and anxiety) (9, 10). In addition, dyslipidemia may decrease the quality of life in patients with MI. Recently, more and more studies have reported HRQoL in patients with MI (11–14), even patients with MI subtypes were also involved (15), and some studies have also investigated the factors that influence the quality of life of patients with MI (16–18). However, most of them only involved sociodemographic and clinical related factors, and the measurement of quality of life in patients with MI combined with dyslipidemia and related factors study had not been observed.

Therefore, hospital case data and questionnaire data were used in this study to evaluate the quality of life based on health preferences of Chinese patients with MI combined with dyslipidemia and to explore factors associated with quality of life. The results of this study will fill in the data gaps, provide real-world evidence for the measurement of QALY of this disease, and help to conduct economic research on relevant interventions. In the meanwhile, the data on quality of life and its relationship with related factors will help healthcare professionals adjust and improve care strategies for patients with this disease and help policymakers evaluate the effectiveness of policies from the perspective of patient-centered care.

## Methods

### Data Source and Collection

Data from this study were collected from a multi-center retrospective study of Chinese patients with MI combined with dyslipidemia. We divided mainland China into five geographical regions: East, West, South, North, and Central, and selected at least one sample hospital from each region. Therefore, six Grade-A tertiary hospitals in mainland China that have a sound electronic case data platform and agree to participate in this study were selected as sample centers. Patients who met inclusion-exclusion criteria in six sample centers were enrolled in the study.

The basic information and clinical characteristics of patients were obtained from electronic medical records. We also conducted a cross-sectional study to collect information on patients' quality of life and lifestyle. From May to July, 2019, patients meeting the requirements were invited to the sample centers for face-to-face questionnaire survey. The ethical evaluation of this study was conducted by the ethics committees of all the six participating hospitals.

### Population

We screened patients according to the hospital's electronic medical record data and we did telephone interview to determine whether patients agreed to enter the study. The patients who met the inclusion and exclusion criteria were asked whether they could go to sample hospitals for a questionnaire survey and whether they were willing to participate in the study.

Inclusion criteria: (1) Patients who were admitted to the hospital for AMI between January 1, 2016, and December 31, 2016, and the earliest hospitalization of a patient with myocardial infarction is regarded as “index hospitalization.” There were no restrictions on whether the patient had complications or had a first episode of myocardial infarction in “index hospitalization”; (2) Patients did not die before face-to-face questionnaire survey; (3) Patients were taking lipid-regulating drugs, or the first blood lipid test showed LDL-C ≥1.8 mmol/L during the “index hospitalization.”

Exclusion criteria: (1) Patients participated in interventional clinical trials after “index hospitalization”; (2) There is a barrier to communication between investigators and the patient or family (if the patient has died).

### Health-Related Quality of Life Measurement

HRQoL was measured using the EuroQoL three-dimensional scale (EQ-5D-3L). EQ-5D-3L is one of the most widely used universal scales to describe and evaluate health status, and its score can be used to calculate QALY. EQ-5D-3L has five dimensions: mobility (MO), self-care (SC), usual activities (UA), pain/discomfort (PD), and anxiety/depression. Each dimension is divided into three levels: unlimited, moderately limited, and completely limited, representing the three health states of each dimension, respectively (19). There are up to 243 combinations of all health states in the five dimensions, and each combination has a corresponding value. The Chinese tariff (20) was adopted in this study, with a value range of −0.149-1. −0.149 represents death and 1 represents complete health.

### Statistical Analysis

Descriptive statistics were applied to baseline characteristics. The mean value and standard deviation of EQ-5D-3L utility value were reported and stratified by gender. Considering the non-normal distribution of EQ-5D index scores (Shapiro-Wilk test, *P* < 0.05), Kruskal-Wallis was used to test the significance of EQ-5D score among grouping variables.

To perform multivariate and correlation analysis, the variables expected to be related to HRQoL were divided into three categories: sociological characteristics (Gender, Age, BMI, Medical insurance, Education status, Marital status, Employment status, Income level), lifestyle (Smoking, Drinking, Diet: whole Grains, Diet: high-fat and high-cholesterol, Sports) and clinical characteristics (Family history of dyslipidemia, Medical history: MI, hypertension, type 2 diabetes, the disorder of lipid metabolism, post PCI, peripheral artery disease). A multivariate Tobit regression model was used to evaluate the relationship between MI combined with dyslipidemia and HRQoL. The model adjusted for patients' sociological characteristics, lifestyle, and clinical characteristics, and was grouped by gender. A Tobit regression model is a truncated model, which can be used for upper or lower truncated problems in cross-sectional studies. The correct Tobit model allows nursing researchers to improve the estimate of bias coefficients through review when measures of health status are limited by limited data (21).

All data were analyzed using Stata SE 15 (Stata Software, StataCorp), and *P* ≤ 0.05 was considered a statistically significant level.

## Results

### Baseline Characteristics

A total of 756 patients responded to the survey. All patients agreed to participate in the study and received informed consent. A total of 756 respondents (613 male and 143 female) completed the survey. Table 1 presents the respondents' general characteristics and means EQ-5D index score. The average age of the 756 patients was 60.45 (SD: 10.88) years old, and most of the patients were >50 and ≤70. Nearly 50% of the patients' BMI was >23.9 and ≤27.9, and 89.42% of the patients had basic medical insurance. Additionally, 74.07% of the patients had medium education and 34.1% of the patients had retired. 58.33% of the patients had hypertension. The average EQ-5D score of all the patients was 0.95 (SD: 0.11). Respondents who were male, who were younger, who were highly educated, married, high income (≥5,500 yuan/month), who often smoke, often drink, often control High-fat and high-cholesterol, often do sport, who had a disorder of lipid metabolism, who had medium and great medication adherence obtained higher index scores.

**Table 1:** Baseline information and EQ-5D index score of all patients.

**Variable**	**Overall (*n* = 756) [*n*(%)]**	**Male [*n*(%)]**	**Female [*n*(%)]**	**EQ-5D index score (SD)**	***P*-value**
Overall	756 (100.00)	613 (100.00)	143 (100.00)	0.95 (0.11)	
Gender					0.000^***^
Female	143 (18.92)		143 (100.00)	0.91 (0.17)	
Male	613 (81.08)	613 (100.00)		0.96 (0.10)	
Age^#^	60.45 (SD:10.88)	59.53 (SD:10.63)	64.39 (SD:11.09)		
Age classification (year)					0.000^***^
≤50	121 (16.01)	113 (18.43)	8 (5.59)	0.97 (0.09)	
>50, ≤60	238 (31.48)	194 (31.65)	44 (30.77)	0.96 (0.09)	
>60, ≤70	272 (35.98)	220 (35.89)	52 (36.36)	0.95 (0.10)	
>70	125 (16.53)	86 (14.03)	39 (27.27)	0.90 (0.18)	
BMI (kg/m2)					0.573
≤18.4	19 (2.51)	14 (2.28)	5 (3.50)	0.96 (0.08)	
>18.4, ≤23.9	252 (33.33)	202 (32.95)	50 (34.97)	0.95 (0.10)	
>23.9, ≤27.9	380 (50.26)	316 (51.55)	64 (44.76)	0.95 (0.12)	
>27.9	105 (13.89)	81 (13.21)	24 (16.78)	0.94 (0.14)	
Medical insurance					0.068
Basic medical insurance system^a^	676 (89.42)	546 (89.07)	130 (90.91)	0.95 (0.11)	
Other medical insurance system	45 (5.95)	38 (6.20)	7 (4.90)	0.96 (0.10)	
Uninsured	35 (4.63)	29 (4.73)	6 (4.20)	0.91 (0.15)	
Education status^b^					0.015^*^
Primary education	105 (13.89)	60 (9.79)	45 (31.47)	0.93 (0.11)	
Medium education	560 (74.07)	469 (76.51)	91 (63.64)	0.95 (0.12)	
High education	91 (12.04)	84 (13.70)	7 (4.90)	0.96 (0.09)	
Marital status					0.009^**^
Current single^c^	44 (5.82)	30 (4.89)	14 (9.79)	0.91 (0.12)	
Married	712 (94.18)	583 (95.11)	129 (90.21)	0.95 (0.11)	
Employment status^d^					0.076
Formal wage	144 (19.05)	137 (22.35)	7 (4.90)	0.97 (0.09)	
Non-formal wage	91 (12.04)	60 (9.79)	31 (21.68)	0.94 (0.12)	
Retired	503 (66.53)	400 (65.25)	103 (72.03)	0.96 (0.89)	
Other	18 (2.38)	16 (2.61)	2 (1.40)	0.95 (0.12)	
Income level (yuan)^e^					0.000^***^
<2,400	187 (24.74)	132 (21.53)	55 (38.46)	0.96 (0.08)	
≥2,400, <4,000	186 (24.60)	140 (22.84)	46 (32.17)	0.93 (0.12)	
≥4,000, <5,500	191 (25.26)	162 (26.43)	29 (20.28)	0.93 (0.16)	
≥5,500	192 (25.40)	179 (29.20)	13 (9.09)	0.97 (0.07)	
Smoking					0.037^*^
No smoking history/give up smoking	496 (65.61)	360 (58.73)	136 (95.10)	0.94 (0.13)	
Sometimes	88 (11.64)	87 (14.19)	1 (0.70)	0.96 (0.09)	
Often	172 (22.75)	166 (27.08)	6 (4.20)	0.97 (0.08)	
Drinking					0.006^**^
No drinking history/give up drinking	515 (68.12)	379 (61.83)	136 (95.10)	0.94 (0.13)	
Sometimes	186 (24.60)	179 (29.20)	7 (4.90)	0.96 (0.08)	
Often	55 (7.28)	55 (8.97)	0 (0.00)	0.97 (0.07)	
Diet (whole grains)					0.070
Often	534 (70.63)	423 (69.00)	111 (77.62)	0.95 (0.11)	
Sometimes	214 (28.31)	183 (29.85)	31 (21.68)	0.94 (0.12)	
Never	8 (1.06)	7 (1.14)	1 (0.70)	0.95 (0.07)	
Diet (high-fat and high-cholesterol)					0.000^***^
Often control	550 (72.75)	441 (71.94)	109 (76.22)	0.95 (0.11)	
Sometimes control	170 (22.49)	142 (23.16)	28 (19.58)	0.93 (0.12)	
Never control	36 (4.76)	30 (4.89)	6 (4.20)	0.91 (0.13)	
Sports					0.000^***^
Often	467 (61.77)	377 (61.50)	90 (62.94)	0.96 (0.09)	
Sometimes	178 (23.54)	151 (24.63)	27 (18.88)	0.95 (0.09)	
Never	111 (14.68)	85 (13.87)	26 (18.18)	0.87 (0.19)	
Family history of dyslipidemia					0.075
No	479 (63.36)	388 (63.30)	91 (63.64)	0.95 (0.12)	
Yes	277 (36.64)	225 (36.70)	52 (36.36)	0.94 (0.11)	
Medical history					
Myocardial infarction (MI)^f^					0.374
No	718 (94.97)	581 (94.78)	137 (95.80)	0.95 (0.11)	
Yes	38 (5.03)	32 (5.22)	6 (4.20)	0.94 (0.11)	
Hypertension					0.327
No	315 (41.67)	259 (42.25)	56 (39.16)	0.95 (0.12)	
Yes	441 (58.33)	354 (57.75)	87 (60.84)	0.94 (0.11)	
Type 2 diabetes					0.258
No	533 (70.50)	437 (71.29)	96 (67.13)	0.95 (0.11)	
Yes	223 (29.50)	176 (28.71)	47 (32.87)	0.94 (0.13)	
Disorder of lipid metabolism					0.039^*^
No	520 (68.78)	426 (69.49)	94 (65.73)	0.95 (0.11)	
Yes	236 (31.22)	187 (30.51)	49 (34.27)	0.95 (0.13)	
Post PCI					0.524
No	222 (29.37)	172 (28.06)	50 (34.97)	0.95 (0.12)	
Yes	534 (70.63)	441 (71.94)	93 (65.03)	0.95 (0.11)	
Peripheral artery disease					0.115
No	656 (86.77)	538 (87.77)	118 (82.52)	0.95 (0.11)	
Yes	100 (13.23)	75 (12.23)	25 (17.48)	0.93 (0.13)	

*PCI, percutaneous coronary intervention; BMI, Body mass index; In BMI, each category includes an upper limit and does not include a lower limit*.

# *Mean and standard deviation were reported*.

a *Basic medical insurance = BMISUE + BMISUR + NRCMS. BMISUE, basic medical insurance system for urban employees; BMISUR, basic medical insurance system for urban residents; NRCMS, the new rural cooperative medical system*.

b *Education status: primary education = not graduated from primary school + primary school; medium education = Junior high school + high school + technical secondary school/junior college graduate; high education = Bachelor + master + doctor*.

c *Current single = unmarried + divorce + death of a spouse*.

d *Employment status: formal wage = formal employees + individuals and freelancers; non-formal wage = Farming + unemployed*.

e *Income is grouped according to quartile*.

f *Patients who experienced MI before index hospitalization*.

* *P < 0.05*;

** *P < 0.01*;

*** *P < 0.001*.

**Figure 1:**
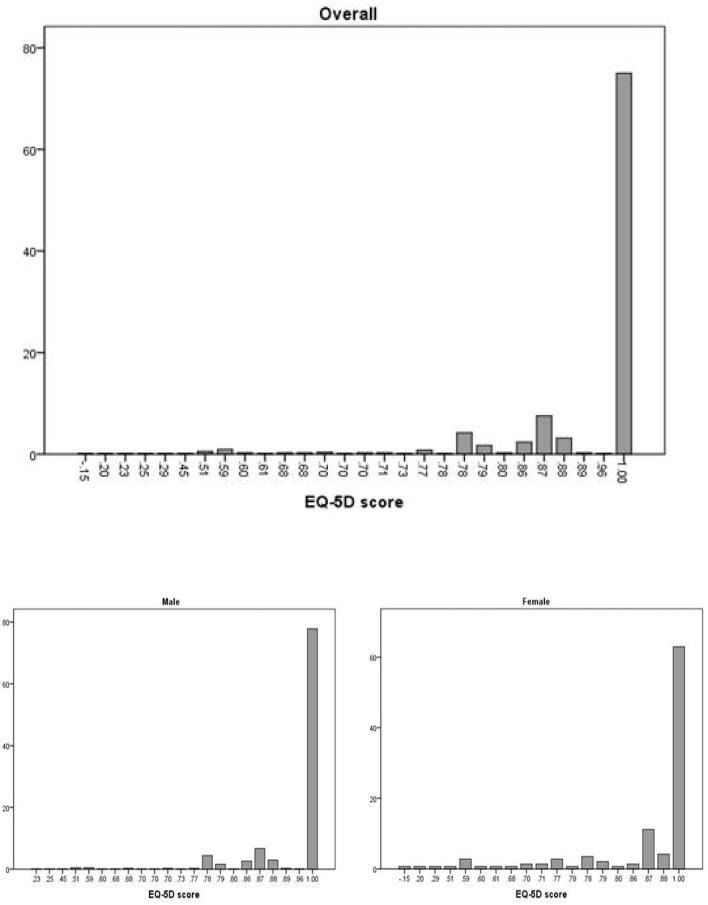
The distribution of the EQ-5D index score.

Figure 1 presents the distribution of the EQ-5D index score. Overall, the distribution was extremely uneven, with a significant difference between 1 and 0.96.

### The Associated Factors of HRQoL

The Results of the Tobit regression for the EQ-5D score are shown in Table 2. Tobit regression model shows that compared with patients aged >18 and ≤ 50 years old, patients aged >70 years old (coefficient = −0.17, *P* < 0.01) had poorer HRQoL; There was statistical significance between the increase in the frequency of high-fat and high-cholesterol food consumption and the decrease in the index score (sometimes control: coefficient = −0.09, *P* < 0.01; Never control: coefficient = −0.17, *P* < 0.01); Patients who never exercise (coefficient = −0.22, *P* < 0.001) had worse HRQoL than those who often exercise. Compared with patients without a family history of dyslipidemia, patients with a family history of dyslipidemia (coefficient = −0.07, *P* < 0.05) had worse HRQoL. Compared with patients without peripheral artery disease, patients with this disease (coefficient = −0.08, *P* < 0.05) had poorer HRQoL.

**Table 2:** Tobit regression for EQ-5D score.

**Variable**		**Overall**		**Male**		**Female**	
		**Coefficient (se)**	***P*-value^#^**	**Coefficient (se)**	***P*-value^#^**	**Coefficient (se)**	***P*-value^#^**
Gender	Male	Ref					
	Female	−0.07 (0.04)	0.053				
Age classification (year)	>18, ≤50	Ref		Ref		Ref	
	>50, ≤60	−0.07 (0.05)	0.135	−0.09 (0.05)	0.080	−0.05 (0.20)	0.810
	>60, ≤70	−0.07 (0.06)	0.246	−0.05 (0.06)	0.371	−0.12 (0.19)	0.531
	>70	−0.17 (0.06)	0.008^**^	−0.13 (0.07)	0.046^*^	−0.20 (0.20)	0.320
BMI (kg/m^2^)	≤18.4	Ref		Ref		Ref	
	>18.4, ≤23.9	−0.13 (0.08)	0.125	−0.33 (0.11)	0.002^**^	0.14 (0.13)	0.296
	>23.9, ≤27.9	−0.13 (0.08)	0.100	−0.33 (0.10)	0.001^**^	0.12 (0.13)	0.381
	>27.9	−0.15 (0.09)	0.088	−0.32 (0.11)	0.003^**^	0.12 (0.15)	0.420
Medical insurance	Basic medical insurance system^a^	Ref		Ref		Ref	
	Other medical insurance system	−0.01 (0.07)	0.918	0.04 (0.07)	0.584	−0.06 (0.15)	0.671
	Uninsured	−0.12 (0.07)	0.098	−0.14 (0.08)	0.084	−0.24 (0.14)	0.088
Education status^b^	Primary education	Ref		Ref		Ref	
	Medium education	0.03 (0.04)	0.535	0.03 (0.05)	0.501	−0.03 (0.09)	0.732
	High education	0.04 (0.06)	0.519	−0.00 (0.07)	0.983	1.83 (0.31)	0.000^***^
Marital status	Current single^c^	Ref		Ref		Ref	
	Married	0.08 (0.05)	0.068	0.10 (0.06)	0.066	0.06 (0.08)	0.496
Employment status^d^	Formal wage	Ref		Ref		Ref	
	Non-formal wage	0.04 (0.07)	0.580	0.04 (0.07)	0.617	−0.24 (0.26)	0.368
	Retired	0.05 (0.05)	0.332	0.05 (0.05)	0.283	−0.28 (0.24)	0.236
	Other	−0.01 (0.10)	0.915	0.03 (0.11)	0.804	−0.99 (0.30)	0.001^**^
Income level (yuan)^e^	<2,400	Ref		Ref		Ref	
	≥2,400, <4,000	−0.09 (0.05)	0.060	−0.07 (0.05)	0.203	−0.12 (0.09)	0.175
	≥4,000, <5,500	−0.08 (0.05)	0.105	−0.05 (0.05)	0.301	−0.15 (0.10)	0.147
	≥5,500	0.07 (0.05)	0.204	0.08 (0.06)	0.152	0.02 (0.17)	0.911
Smoking	No smoking history/give up smoking	Ref		Ref		Ref	
	Sometimes	0.02 (0.05)	0.742	0.01 (0.05)	0.842	1.29 (0.22)	0.000^***^
	Often	0.06 (0.04)	0.122	0.05 (0.04)	0.202	−0.15 (0.14)	0.295
Drinking	No drinking history/give up drinking	Ref		Ref		Ref	
	Sometimes	0.05 (0.04)	0.167	0.07 (0.04)	0.069	−0.02 (0.16)	0.908
	Often	0.08 (0.06)	0.163	0.09 (0.06)	0.141		
Diet (Whole Grains)	Often	Ref		Ref		Ref	
	Sometimes	−0.03 (0.03)	0.410	−0.02 (0.03)	0.578	−0.12 (0.08)	0.102
	Never	−0.01 (0.10)	0.940	−0.05 (0.10)	0.639	1.19 (0.27)	0.000^***^
Diet (High-fat and high-cholesterol)	Often control	Ref		Ref		Ref	
	Sometimes control	−0.09 (0.03)	0.005^**^	−0.07 (0.04)	0.044^*^	−0.17 (0.07)	0.018^*^
	Never control	−0.17 (0.05)	0.001^**^	−0.15 (0.06)	0.012^*^	−0.07 (0.12)	0.582
Sports	Often	Ref		Ref		Ref	
	Sometimes	−0.02 (0.04)	0.667	−0.02 (0.04)	0.633	−0.03 (0.09)	0.729
	Never	−0.22 (0.04)	0.000^***^	−0.19 (0.04)	0.000^***^	−0.30 (0.08)	0.001^**^
Family history of dyslipidemia	No	Ref		Ref		Ref	
	Yes	−0.07 (0.03)	0.015^*^	−0.08 (0.03)	0.011^*^	0.09 (0.08)	0.263
Medical history							
Myocardial infarction (MI)^f^	No	Ref		Ref		Ref	
	Yes	−0.07 (0.06)	0.256	−0.06 (0.07)	0.373	−0.09 (0.14)	0.514
Hypertension	No	Ref		Ref		Ref	
	Yes	0.01 (0.03)	0.821	−0.03 (0.03)	0.400	0.18 (0.07)	0.006^**^
Type 2 diabetes	No	Ref		Ref		Ref	
	Yes	−0.01 (0.03)	0.687	−0.03 (0.03)	0.323	0.02 (0.07)	0.726
Disorder of lipid metabolism	No	Ref		Ref		Ref	
	Yes	0.06 (0.03)	0.080	0.09 (0.04)	0.019^*^	−0.03 (0.06)	0.636
Post PCI	No	Ref		Ref		Ref	
	Yes	−0.03 (0.03)	0.272	−0.02 (0.03)	0.580	−0.11 (0.07)	0.114
Peripheral artery disease	No	Ref		Ref		Ref	
	Yes	−0.08 (0.04)	0.048^*^	−0.05 (0.04)	0.311	−0.12 (0.07)	0.087

*Se, robust standard error; PCI, percutaneous coronary intervention; BMI, Body mass index; In BMI, each category includes an upper limit and does not include a lower limit*.

# *P-value are numbers in robust standard error*.

a *Basic medical insurance = BMISUE + BMISUR + NRCMS. BMISUE, basic medical insurance system for urban employees; BMISUR, basic medical insurance system for urban residents; NRCMS, the new rural cooperative medical system*.

b *Education status: primary education = not graduated from primary school + primary school; medium education = Junior high school + high school + technical secondary school/junior college graduate; high education = Bachelor + master + doctor*.

c *Current single = unmarried + divorce + death of a spouse*.

d *Employment status: formal wage = formal employees + individuals and freelancers; non-formal wage = Farming + unemployed*.

e *Income is grouped according to quartile*.

f *Patients who experienced MI before index hospitalization*.

* *P < 0.05*;

** *P < 0.01*;

*** *P < 0.001*.

For the male, different from all patients, the patients with a Disorder of lipid metabolism (coefficient = 0.09, *P* < 0.05) had better HRQoL than those without, and no significant change was observed in peripheral artery disease. In addition, male patients showed a significant difference in BMI. Compared with patients with BMI ≤ 18.4, patients with higher BMI (>18.4, ≤23.9: coefficient = −0.33, *P* < 0.01; >23.9, ≤27.9: coefficient = −0.33, *P* < 0.01; >27.9: coefficient = −0.32, *P* < 0.01) had poorer HRQoL.

For the female, patients with Hypertension have better HRQoL than those without (coefficient = 0.18, *P* < 0.01), and no significant change was observed in peripheral artery disease, family history, and never control high-fat and high-cholesterol food. In addition, patients with higher education (coefficient = 1.83, *P* < 0.001) had better HRQoL than patients with lower education; Patients with other employment types (coefficient = −0.99, *P* < 0.01) had worse HRQoL than those with formal jobs. Patients who smoked occasionally (coefficient = 1.29, *P* < 0.001) had better HRQoL than those who did not currently smoke. Patients who never ate Whole Grains Food (coefficient = 1.19, *P* < 0.001) had better HRQoL than those who often eat Whole Grains Food regularly.

## Discussion

This study first reported the quality of life and related factors of patients in MI combined with dyslipidemia. Our study used EQ-5D to determine the quality of life of patients with MI combined with dyslipidemia and used the Tobit model to evaluate the influencing factors of the index score. This study fills the gap of HRQoL and its influencing factors in these patients.

In our study, the mean EQ-5D-3L index score for all patients was 0.95. There are many studies on the quality of life of patients with MI, but most of them used scales that cannot obtain the utility value directly. Lee et al. adopted the Coronary Revascularization Outcome Questionnaire (CROQ) to measure the HRQoL of Korean patients with MI (22). Ul-Haq et al. used the General Health Questionnaire (GHQ), Self-rated Health (SRH), and one post-MI specific tool (MacNew QLMI) Celia to measure HRQoL of Pakistani with MI (23). Wlodarczyk et al. used the Nottingham Health Profile (NHP) to measure the HRQoL of Polish with MI (24). Munyombwe et al. used EQ-5D-3L to measure the HRQoL of British with MI. The mean index score was 0.78 (SD: 0.3) (25). In contrast, the Quality of life in our study was higher among patients with MI combined with dyslipidemia, which may be correlated with the year of measurement and individual differences and nursing differences. Since HRQoL is one of the key data points for cost-effectiveness approaches, more research should focus on determining the regional and global quality of life index scores for this population.

In our study, like the results of other Quality of life studies of MI patients, the female in our study had a lower HRQoL than the male (24, 25). Female and male had different predictors, unlike previous study (26), highly educated women had higher quality of life, but there were no significant differences in marital status. In addition, our study also included lifestyle variables. The results showed that there were significant differences in quality of life among females on factors such as smoking and dietary fiber diet. We observed that patients who sometimes smoke had a higher quality of life than current non-smokers, which may be related to the “smokers' paradox.” Several studies have found that smokers had a lower mortality rate after AMI than non-smokers (27, 28). And patients who survived after MI were more likely to be smokers (29). The reduction of mortality may be related to the decrease of MI recurrence, and the reduction of recurrence may increase the patient's HRQoL. In terms of diet, patients may consume high-fat food while eating dietary fiber. Hyperlipidemia may increase the risk of MI recurrence and affect patients physically and mentally, thus reducing the quality of life.

For all patients, analysis of influencing factors showed that a healthy lifestyle (exercise, frequent control high-fat consumption) improved quality of life. Exercise and control of a high-fat diet have synergistic effects on fat consumption and fat intake, respectively. The reduction of fat may reduce the risk of hyperlipidemia and thus improve the quality of life. In addition, exercise boosts dopamine production, and physical activity is associated with the improvement of depression and anxiety (30, 31). Multiple studies have indicated that depression or anxiety was an overseen complication in MI patients (32–34). The above analysis showed that exercise helps improve the HRQoL of MI patients. These also suggest that in the treatment of patients with MI combined with dyslipidemia, the improvement of lifestyle should be strengthened, and the patient's compliance with a healthy lifestyle should be improved, as well as the control of fat level.

There are some limitations to this study. First, this study is a cross-sectional study of a retrospective analysis, so it is unable to explore the long-term changes in quality of life and the changes in related predictors of patients with MI combined with dyslipidemia, which is also the future direction of this study. Secondly, the EQ-5D-3L scale was adopted in this study to measure the quality of life of patients, which may be influenced by the ceiling effect compared with the EQ-5D-5L scale. Thirdly, patients in this study were from six tertiary hospitals, and patients in secondary and primary hospitals were not included, so there may be a certain selection bias.

## Conclusion

Dyslipidemia is one of the risk factors of MI. Although there have been many studies on the quality of life of patients with MI, there is no study to measure the quality of life of patients with MI combined with dyslipidemia. This research adopts the EQ-5D-3L to measure the HRQoL of the patients with MI combined with dyslipidemia, at the same time analyze the predictive factors of HRQoL from the sociology characteristics, lifestyle, and clinical characteristics. The results of this study fill in the data gaps in the field of related diseases and provide real-world data for cost-effectiveness analysis. The study found that the quality of life of female was lower than male, and female had more predictors; In addition, for all patients, we should pay more attention to the post-discharge care of the elderly group, increase the advocacy and suggestions on healthy lifestyle (fat control) of the patients to improve the quality of life of these patients.

## Data Availability Statement

The datasets presented in this article are not readily available because sharing of raw data would be contingent on approval from the research ethics office because of the ethics consideration. Requests to access the datasets should be directed to lihongchao@cpu.edu.cn.

## Ethics Statement

The studies involving human participants were reviewed and approved by Peking University First Hospital; Wuhan Asia Heart Hospital; Guangdong Provincial People's Hospital; Nanjing Drum Tower Hospital; Sichuan Provincial People's Hospital; Zhongshan Hospital, Fudan University. The patients/participants provided their written informed consent to participate in this study.

## Author Contributions

AM, HL, and PC designed this study. Data were collected and managed by YZ, XS, JC, BX, JT, and ZW. MZ performed the statistics using software and drafted the manuscript. AM and HL supervised the data analysis and proposed suggestions for revising the manuscript. All authors critically reviewed the manuscript, approved the final version of the manuscript, and agree to be accountable for the content of the work.

## Funding

The authors declare that this study received funding from Amgen Biotech Consultation (Shanghai) Co., Ltd. The work was supported under Contract No. 2017-business 39.

## Conflict of Interest

Amgen Biotech Consultation (Shanghai) Co., Ltd partly sponsored this study. However, The funder had no role in study design, data collection and analysis, and preparation of the manuscript. This study does not involve any specific products. The authors declare that the research was conducted in the absence of any commercial or financial relationships that could be construed as a potential conflict of interest.

## Publisher's Note

All claims expressed in this article are solely those of the authors and do not necessarily represent those of their affiliated organizations, or those of the publisher, the editors and the reviewers. Any product that may be evaluated in this article, or claim that may be made by its manufacturer, is not guaranteed or endorsed by the publisher.
